# Evaluation of Maternal Infection During Pregnancy and Childhood Leukemia Among Offspring in Denmark

**DOI:** 10.1001/jamanetworkopen.2023.0133

**Published:** 2023-02-20

**Authors:** Jian-Rong He, Yongfu Yu, Fang Fang, Mika Gissler, Per Magnus, Krisztina D. László, Mary H. Ward, Ora Paltiel, Gabriella Tikellis, Milena Maria Maule, Xiu Qiu, Jiangbo Du, Unnur Anna Valdimarsdóttir, Kazem Rahimi, Joseph L. Wiemels, Martha S. Linet, Jane E. Hirst, Jiong Li, Terence Dwyer

**Affiliations:** 1 Division of Birth Cohort Study, Guangzhou Women and Children’s Medical Center, Guangzhou Medical University, Guangzhou, China; 2 Nuffield Department of Women’s and Reproductive Health, University of Oxford, Oxford, United Kingdom; 3 Department of Biostatistics, School of Public Health, Fudan University, Shanghai, China; 4 Key Laboratory of Public Health Safety of the Ministry of Education, Fudan University, Shanghai, China; 5 Department of Clinical Medicine, Aarhus University, Aarhus, Denmark; 6 Department of Clinical Epidemiology, Aarhus University, Aarhus, Denmark; 7 Institute of Environmental Medicine, Karolinska Institute, Stockholm, Sweden; 8 Department of Molecular Medicine and Surgery, Karolinska Institute, Solna, Sweden; 9 Academic Primary Health Care Centre, Region Stockholm, Sweden; 10 Department of Child Psychiatry, Turku University Hospital, Turku University, Turku, Finland; 11 Department of Knowledge Brokers, Finnish Institute for Health and Welfare, Helsinki, Finland; 12 Centre for Fertility and Health, Norwegian Institute of Public Health, Oslo, Norway; 13 Department of Global Public Health, Karolinska Institute, Stockholm, Sweden; 14 Occupational and Environmental Epidemiology Branch, Division of Cancer Epidemiology and Genetics, National Cancer Institute, National Institutes of Health, Rockville, Maryland; 15 Braun School of Public Health and Community Medicine, Hadassah Medical Organization, Faculty of Medicine, Hebrew University of Jerusalem, Jerusalem, Israel; 16 Murdoch Children’s Research Institute, Royal Children’s Hospital, University of Melbourne, Melbourne, Australia; 17 Cancer Epidemiology Unit, Department of Medical Sciences, University of Turin, Turin, Italy; 18 Azienda Ospedaliera Universitaria Città della Salute e della Scienza, Turin, Italy; 19 Department of Epidemiology, School of Public Health, Nanjing Medical University, Nanjing, China; 20 Center of Public Health Sciences, University of Iceland, Reykjavik, Iceland; 21 Department of Medical Epidemiology and Biostatistics, Karolinska Institute, Stockholm, Sweden; 22 Center for Genetic Epidemiology, University of Southern California, Los Angeles; 23 Division of Cancer Epidemiology and Genetics, National Cancer Institute, National Institutes of Health, Bethesda, Maryland; 24 George Institute for Global Health, London, United Kingdom; 25 Clinical Sciences Theme, Heart Group, Murdoch Children’s Research Institute, Melbourne, Australia; 26 Faculty of Medicine, Dentistry and Health Sciences, University of Melbourne, Melbourne, Australia

## Abstract

**Importance:**

Maternal infection is common during pregnancy and is an important potential cause of fetal genetic and immunological abnormalities. Maternal infection has been reported to be associated with childhood leukemia in previous case-control or small cohort studies.

**Objective:**

To evaluate the association of maternal infection during pregnancy with childhood leukemia among offspring in a large study.

**Design, Setting, and Participants:**

This population-based cohort study used data from 7 Danish national registries (including the Danish Medical Birth Register, the Danish National Patient Registry, the Danish National Cancer Registry, and others) for all live births in Denmark between 1978 and 2015. Swedish registry data for all live births between 1988 and 2014 were used to validate the findings for the Danish cohort. Data were analyzed from December 2019 to December 2021.

**Exposures:**

Maternal infection during pregnancy categorized by anatomic locations identified from the Danish National Patient Registry.

**Main Outcomes and Measures:**

The primary outcome was any leukemia; secondary outcomes were acute lymphoid leukemia (ALL) and acute myeloid leukemia (AML). Offspring childhood leukemia was identified in the Danish National Cancer Registry. Associations were first assessed in the whole cohort using Cox proportional hazards regression models, adjusted for potential confounders. A sibling analysis was performed to account for unmeasured familial confounding.

**Results:**

This study included 2 222 797 children, 51.3% of whom were boys. During the approximately 27 million person-years of follow-up (mean [SD], 12.0 [4.6] years per person), 1307 children were diagnosed with leukemia (ALL, 1050; AML, 165; or other, 92). Children born to mothers with infection during pregnancy had a 35% increased risk of leukemia (adjusted hazard ratio [HR], 1.35 [95% CI, 1.04-1.77]) compared with offspring of mothers without infection. Maternal genital and urinary tract infections were associated with a 142% and 65% increased risk of childhood leukemia, with HRs of 2.42 (95% CI, 1.50-3.92) and 1.65 (95% CI, 1.15-2.36), respectively. No association was observed for respiratory tract, digestive, or other infections. The sibling analysis showed comparable estimates to the whole-cohort analysis. The association patterns for ALL and AML were similar to that for any leukemia. No association was observed for maternal infection and brain tumors, lymphoma, or other childhood cancers.

**Conclusions and Relevance:**

In this cohort study of approximately 2.2 million children, maternal genitourinary tract infection during pregnancy was associated with childhood leukemia among offspring. If confirmed in future studies, our findings may have implications for understanding the etiology and developing preventive measures for childhood leukemia.

## Key Points

**Question:**

Is maternal infection during pregnancy associated with risk of childhood leukemia among offspring in Denmark?

**Findings:**

In this cohort study of 2 million Danish children, maternal infection during pregnancy was associated with a 35% higher risk of childhood leukemia but not other types of childhood cancer. This association was mostly attributed to genital and urinary tract infections, which were associated with a 142% and 65% increased risk of childhood leukemia, respectively.

**Meaning:**

These findings suggest that certain types of maternal infection during pregnancy were associated with an increased risk of childhood leukemia among offspring.

## Teaser

This cohort study used national registry data for all children born in Denmark between 1978 to 2015 to examine the association of maternal infection during pregnancy with childhood leukemia among offspring.

## Introduction

Leukemia is the most common cancer among children.^1^ The etiology of this malignant neoplasm is poorly understood. Childhood leukemia may originate in utero, given that leukemia-related chromosome lesions have been observed at birth.^2^ Previous studies reported that cytokine levels at birth were different for healthy children compared with individuals who developed leukemia in childhood.^3,4^ These findings suggest that immune-related factors during pregnancy may be involved in the development of childhood leukemia.

Maternal infection during pregnancy is common.^5^ Maternal infection may lead to chromosomal^6,7^ or immunological^8^ alterations in the fetus and may be a risk factor for childhood leukemia.^9,10^ In our recent systematic review and meta-analysis, maternal influenza, rubella, and varicella during pregnancy were associated with a higher risk of childhood leukemia.^11^ However, evidence of these associations stems predominantly from case-control studies, which are prone to recall and selection bias. We recently examined the association between maternal infection and childhood leukemia using data on 312 000 mother-child pairs in the International Childhood Cancer Consortium (I4C). We found urinary tract and respiratory tract infections to be associated with an increased risk of childhood leukemia.^12^ Several limitations of the I4C analysis should be acknowledged, such as a small number of leukemia cases, self-reported maternal infection history, and potential confounding by familial factors. To overcome the limitations of previous analyses, we undertook a large population-based cohort study of Danish children to investigate associations between hospital-diagnosed maternal infection during pregnancy categorized by anatomic location and the risk of childhood leukemia.

## Methods

This cohort study was approved by the Data Protection Agency in Denmark and the Regional Ethics Committee in Stockholm, Sweden. Per Danish and Swedish laws, no informed consent was required for register-based studies. This study followed the Strengthening the Reporting of Observational Studies in Epidemiology (STROBE) reporting guideline.

### Study Population

This study included all live births from 1978 to 2015 in Denmark.^13^ We excluded 78 347 nonsingleton births, 2111 children with Down syndrome, and 47 565 mother-child pairs without data on key covariates, resulting in 2 222 797 children included in the final analysis (eFigure 1 in Supplement 1). We linked data from 7 Danish national registries (including the Danish Medical Birth Register, the Danish National Patient Registry, the Danish National Cancer Registry, and others) using unique personal identification numbers (eMethods 1 and eTable 1 in Supplement 1).

### Outcome Ascertainment

Childhood cancers were identified through linkage of the cohort (aged <15 years) to the Danish National Cancer Registry using *International Classification of Diseases, Eighth Revision* (*ICD-8*) and *International Statistical Classification of Diseases and Related Health Problems, Tenth Revision* (*ICD-10*) codes (eTable 2 in Supplement 1). The completeness of cancer ascertainment from the Danish Cancer Registry is high (95%-98%).^14^ The primary outcome was any childhood leukemia. Secondary outcomes were acute lymphoblastic leukemia (ALL) and acute myeloid leukemia (AML). Other childhood cancers, including brain tumors, lymphoma, and other cancers, were analyzed in a sensitivity analysis to investigate whether any associations found with maternal infection were specific to childhood leukemia. All outcomes are time-to-event variable, with the time to event calculated as the interval from birth until the first diagnosis of cancer or censoring (last record linkage, death, emigration, or 15th birthday), whichever came first.

### Exposure Assessment

Maternal infection during pregnancy was identified in the Danish National Patient Registry based on inpatient diagnoses (all study years) and outpatient diagnoses (since 1995).^15^ We used *ICD* codes for 386 infectious diseases to define the infection exposure (eTable 3 in Supplement 1). Exposure variables were as follows: (1) any infection and (2) infections by site, including respiratory tract, genitourinary tract (ie, urinary and genital tract), digestive system, and other infections. Sexually transmitted infection was also considered as a separate exposure variable, as a previous study reported associations with childhood leukemia.^16^ Maternal infection during pregnancy was defined as any diagnosis of infection between the start and end dates of the pregnancy.

### Covariates and Comorbidities

Information on covariates was obtained from various registries (eTable 1 in Supplement 1). The following maternal variables were included in the models for adjustment: age at childbirth, education level (≤9, 10-14, or ≥15 years), parity (primipara or multipara), cohabitation during pregnancy (yes or no), diabetes during pregnancy (yes or no; including prepregnancy diabetes and gestational diabetes), year of delivery (1978-1990, 1991-2000, 2001-2010, or 2011-2015), and season of delivery (spring, summer, autumn, or winter).

In a sensitivity analysis, we additionally adjusted for the following maternal comorbidities during pregnancy: hypertensive disorders,^17,18^ anemia,^17^ obstetric hemorrhage,^19^ hyperemesis,^17^ and asthma.^20^ These comorbidities were identified from the National Patient Registry (eTable 4 in Supplement 1).

### Validation Using Swedish Registry Data

We also obtained data on all 2 682 269 live births during 1988 to 2014 in Sweden (eMethods 2 and eFigure 1 in Supplement 1). Similar to Denmark, data on covariates and leukemia outcomes were obtained through the linkage of several Swedish registries. However, for maternal infection assessment, we only obtained a subset of Swedish Patient Register data for patients with specific diagnostic codes. These codes cover certain infectious conditions and noncommunicable diseases (eTable 5 in Supplement 1). A patient with any of these codes was eligible for inclusion in the subset. For the eligible patients, complete diagnostic codes during the same hospital visit were also available. Maternal infection exposure was then assessed in these subset data using the *ICD* codes listed in eTable 3 in Supplement 1; thus, its ascertainment was less complete than that in the Danish data set (details in eMethods 1 and 2 in Supplement 1). Therefore, the Swedish data set was only used as a validation of the main findings in the Danish data set.

### Statistical Analysis

Cox proportional hazards regression models were used to examine the association between maternal infection and childhood cancer outcomes and to calculate hazard ratios (HRs) and 95% CIs. We used log-log survival plots and a test of Schoenfeld residuals to assess the proportional hazards assumption and found no obvious violation. For infections with a positive association with leukemia, we further analyzed the association by child age at leukemia diagnosis (<1, 1-5, or 6-14 years) and by timing of maternal infection (first, second, or third trimester). The absolute risk difference between exposed and unexposed children was calculated using Poisson regression, standardized to the observed distribution of all covariates.

We further conducted a sibling analysis to examine the potential confounding effect of invariant within-family factors (eg, genetic or other familial characteristics).^21^ In this analysis, the association between maternal infection and any leukemia was examined among children from the same mother, by comparing the risk of outcome between the sibling with maternal infection exposure and the sibling without such exposure. Therefore, the shared factors within a family were automatically matched and controlled for statistically. The sibling analysis was conducted using a stratified Cox proportional hazards regression model with a separate stratum for each family identified by the maternal unique identification number. Thus, each family had a distinctive baseline hazard, which reflects the shared familial clustering factors.^22^ Although all children with siblings were included in this analysis, only the sibling pairs with both discordant exposure (ie, 1 child was exposed but the sibling was not) and discordant outcome (ie, 1 child had leukemia but the sibling did not) were informative for the calculation of the effect estimate. The sibling analysis also included the same set of covariates as the whole-cohort analysis.

Subgroup analyses were performed to examine whether the effect estimates differed between the years with and without outpatient data (ie, 1995-2015 vs 1978-2014) or between periods using *ICD-10* (1994 and afterward) or *ICD-8* codes (1978-1993) for hospital diagnosis in Denmark. Data on maternal smoking during early pregnancy and prepregnancy body mass index (BMI) were available only for certain years (ie, 1991-2015 for maternal smoking; 2004-2015 for prepregnancy BMI). To assess potential confounding by these 2 factors, we restricted the analysis to the years when data were available and compared results between models with and without adjustment for the 2 factors. Sensitivity analyses were performed by additionally adjusting for maternal comorbidities during pregnancy or excluding children born preterm. To investigate the potential impact of the clustering effect of children from the same mother on the results, we performed a sensitivity analysis using a robust standard error to calculate the variance of the estimates. We found that results taking into account the clustering effect were very similar to those without considering this clustering effect. Therefore, we reported estimates without taking the clustering effect into account as the primary analysis.

Analyses were performed using Stata, version 14 (StataCorp), and SAS, version 9.4 (SAS Institute). Statistical significance was set as a 2-tailed *P* < .05. Data were analyzed from December 2019 to December 2021.

## Results

### Population Characteristics

This study included 2 222 797 Danish children, with approximately 27 million person-years of follow-up (mean [SD], 12.0 [4.6] years per person); 51.3% were boys and 48.7% were girls. A total of 4362 children were diagnosed with cancer before age 15 years, including 1307 with leukemia (ALL, 1050; AML, 165; or other, 92), 1267 with brain tumor, 224 with lymphoma, and 1564 with other cancers. A total of 81 717 mothers (3.7%) had at least 1 type of infection during pregnancy. Urinary tract infection (37 522 [1.7%]) was the most common, followed by genital tract (14 382 [0.7%]), digestive system (10 097 [0.5%]), and respiratory tract (7318 [0.3%]) infections. Compared with those without infection, mothers with any infection during pregnancy were more likely (1) to be slightly younger and primiparous; (2) to have fewer years of education, higher prepregnancy BMI, and diabetes and to smoke during early pregnancy; and (3) to deliver preterm and low birth weight infants (Table 1).

**Table 1.** Characteristics of Offspring by Maternal Infection During Pregnancy

Characteristic	No. of children (%) (N = 2 222 797)		*P* value
	No maternal infection (n = 2 141 080)	Any maternal infection (n = 81 717)	
Maternal age, y, mean (SD)	28.8 (5.0)	28.1 (5.5)	<.001
Maternal education, y			
≤9	581 763 (27.2)	28 730 (35.2)	<.001
10-14	944 453 (44.1)	33 541 (41.0)	
≥15	614 864 (28.7)	19 446 (23.8)	
Maternal cohabitation			
No	963 439 (45.0)	42 749 (52.3)	<.001
Yes	1 177 641 (55.0)	38 968 (47.7)	
Parity			
Primipara	953 149 (44.5)	41 088 (50.3)	<.001
Multipara	1 187 931 (55.5)	40 629 (49.7)	
Maternal diabetes			
No	2 095 735 (97.9)	78 176 (95.7)	<.001
Yes	45 345 (2.1)	3541 (4.3)	
Smoking during early pregnancy^a^			
No	1 110 991 (79.8)	46 217 (75.0)	<.001
Yes	280 518 (20.2)	15 391 (25.0)	
Missing^b^	58 731	2448	
Prepregnancy body mass index^c^			
Mean (SD)	24.3 (4.8)	24.7 (5.3)	<.001
Missing^b^	36 796	1735	
Child sex			
Male	1 098 379 (51.3)	42 091 (51.5)	.09
Female	1 041 739 (46.7)	39 601 (48.5)	
Missing^b^	962	25	
Preterm birth			
No	1 970 911 (95.4)	72 632 (90.7)	<.001
Yes	95 524 (4.6)	7432 (9.3)	
Missing^b^	74 645	1653	
Birth weight, g			
<2500	79 444 (3.7)	5832 (7.2)	<.001
2500-3999	1 668 646 (78.7)	62 798 (77.5)	
≥4000	371 372 (17.5)	12 423 (15.3)	
Missing^b^	21 618	664	

^a^ Data on maternal smoking were available from 1991 onward.

^b^ Number of children with missing data.

^c^ Data on prepregnancy body mass index (calculated as weight in kilograms divided by height in meters squared) were available from 2004 onward.

### Any Childhood Leukemia

**Figure 1.**
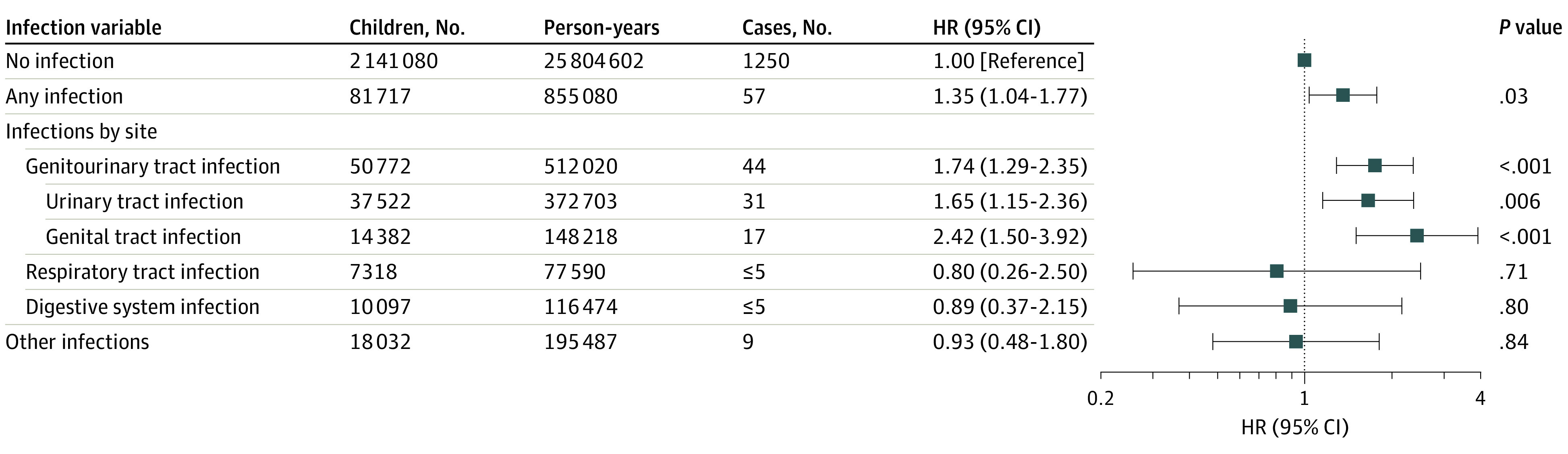
Association of Any Maternal Infection and Specific Infection Types During Pregnancy With Any Childhood Leukemia in Offspring All models were adjusted for maternal age, educational level, parity, cohabitation during pregnancy, any diabetes during pregnancy, birth year, and birth season. All hazard ratios (HRs) were calculated by comparing children with each type of maternal infection exposure vs children without any maternal infection exposure.

After adjustment for confounders, any maternal infection was associated with a 35% increased risk of childhood leukemia (HR, 1.35 [95% CI, 1.04-1.77]; Figure 1). This association was driven by genital tract infection (HR, 2.42 [95% CI, 1.50-3.92]) and urinary tract infection (HR, 1.65 [95% CI, 1.15-2.36]), which were associated with a 142% and 65% increased risk, respectively, of childhood leukemia. Children of women with sexually transmitted infection (HR, 3.13 [95% CI, 1.73-5.67]) were more likely to develop leukemia compared with those not exposed. There was no association between other maternal infections and childhood leukemia. The absolute risk difference between exposed and unexposed children was 1.8 cases (95% CI, 0.01-3.6) for any infection, 3.4 cases (95% CI, 0.4-6.4) for urinary tract infection, and 7.1 cases (95% CI, 1.3-12.0) for genital tract infection per 100 000 person-years.

### Childhood Leukemia Subtypes

Results for ALL and AML were similar to those for any leukemia. Genitourinary tract infection was associated with a higher risk of ALL and AML, whereas no association was observed for other infections (eFigure 2 in Supplement 1).

### Sibling Analysis

Findings from the sibling analysis were similar to those in the whole-cohort analysis (Table 2), indicating that shared familial factors did not substantially contribute to the association between maternal infection and childhood leukemia. For example, the HRs for urinary tract infection were 1.45 (95% CI, 0.80-2.63) in the sibling analysis and 1.65 (95% CI, 1.15-2.36) in the whole-cohort analysis, and the HRs for genital tract infection were 3.93 (95% CI, 1.56-9.90) in the sibling analysis and 2.42 (95% CI, 1.50-3.92) in the whole-cohort analysis. The 95% CIs for the HRs in the sibling analysis were wider, given the much smaller sample size.

**Table 2.** Results of Sibling Analysis for Maternal Infection and Any Childhood Leukemia^a^

Maternal infection during pregnancy	No. of pairs of siblings with discordant exposure and outcome	Hazard ratio (95% CI)
Any	101	1.43 (0.94-2.18)
Genitourinary tract	70	1.76 (1.06-2.90)
Urinary tract	51	1.45 (0.80-2.63)
Genital tract	23	3.93 (1.56-9.90)
Respiratory tract	7	1.54 (0.32-7.33)
Digestive system	16	0.68 (0.22-2.11)
Other	21	0.85 (0.33-2.17)

^a^ All models were adjusted for maternal age, educational level, parity, cohabitation during pregnancy, any diabetes during pregnancy, birth year, and birth season.

### Subgroup and Sensitivity Analyses

**Figure 2.**
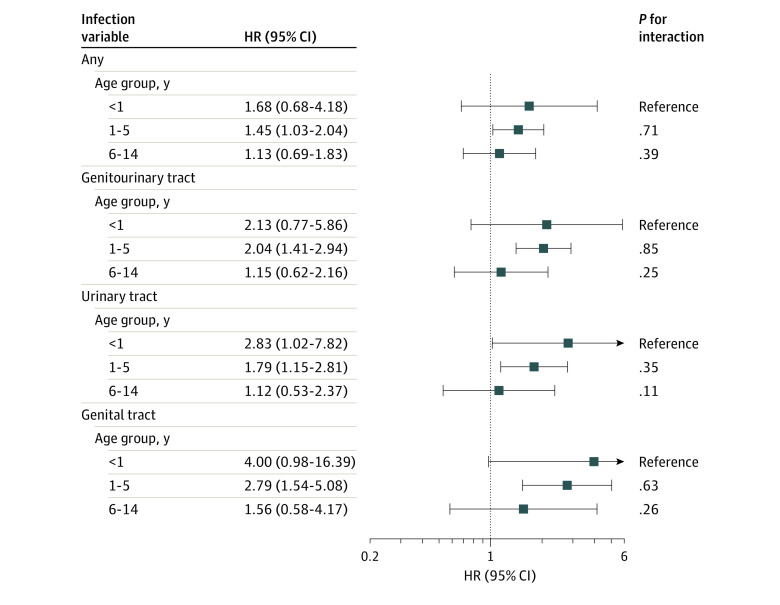
Stratified Association of Maternal Infection for Any Childhood Leukemia by Diagnosis Age All models were adjusted for maternal age, educational level, parity, cohabitation during pregnancy, any diabetes during pregnancy, birth year, and birth season. HR indicates hazard ratio.

For infections showing a positive association with any leukemia, HRs were greater for children aged younger than 1 year and 1 to 5 years compared with those aged 6 to 14 years (Figure 2). However, the differences between age groups were not statistically significant. In addition, maternal infections at different trimesters had similar associations with any leukemia (eTable 6 in Supplement 1).

Associations of maternal infection and any leukemia were similar between the periods with and without outpatient data or between periods using *ICD-10* or *ICD-8* codes in Denmark (eTable 7 in Supplement 1). Similar results were observed between models with and without adjustment for maternal smoking and prepregnancy BMI (eTable 8 in Supplement 1). Adjusting for maternal comorbidities or excluding preterm births did not change the results substantially (eTables 9 and 10 in Supplement 1). Accounting for clustering effects from children with the same mother led to almost identical results as the primary analysis (data not shown).

### Other Childhood Cancers

No association was observed between maternal infection and childhood brain tumors, lymphomas, or other cancers (Table 3). Respiratory tract infection had a high HR for brain tumors (1.93 [95% CI, 0.92-4.05]) and lymphoma (2.76 [95% CI, 0.69-11.13]), but the precision of these estimates was low.

**Table 3.** Association of Maternal Infection During Pregnancy With Other Types of Childhood Cancer^a^

Cancer type, infection	No. of children	Person-years	No. of cases	Hazard ratio (95% CI)	*P* value
Brain tumor					
No	2 141 080	25 804 602	1217	1 [Reference]	NA
Any	81 717	855 080	50	1.24 (0.94-1.65)	.13
Genitourinary tract	50 772	512 020	29	1.21 (0.83-1.75)	.32
Urinary tract	37 522	372 703	21	1.21 (0.78-1.86)	.39
Genital tract	14 382	148 218	9	1.28 (0.67-2.47)	.46
Respiratory tract	7318	77 590	7	1.93 (0.92-4.05)	.08
Digestive system	10 097	116 474	6	1.10 (0.49-2.45)	.82
Other	18 032	195 487	11	1.20 (0.66-2.18)	.54
Lymphoma					
No	2 141 080	25 804 602	213	1 [Reference]	NA
Any	81 717	855 080	11	1.29 (0.70-2.37)	.41
Genitourinary tract	50 772	512 020	7	1.32 (0.62-2.81)	.48
Urinary tract	37 522	372 703	≤5	0.99 (0.37-2.66)	.98
Genital tract	14 382	148 218	≤5	2.20 (0.70-6.89)	.18
Respiratory tract	7318	77 590	≤5	2.76 (0.69-11.13)	.15
Digestive system	10 097	116 474	0	NA^b^	NA
Other	18 032	195 487	≤5	1.09 (0.27-4.38)	.91
Other cancer^c^					
No	2 141 080	25 804 602	1510	1 [Reference]	NA
Any	81 717	855 080	54	1.09 (0.83-1.43)	.55
Genitourinary tract	50 772	512 020	35	1.17 (0.84-1.64)	.35
Urinary tract	37 522	372 703	26	1.21 (0.82-1.78)	.34
Genital tract	14 382	148 218	10	1.14 (0.61-2.13)	.68
Respiratory tract	7318	77 590	≤5	0.45 (0.11-1.78)	.25
Digestive system	10 097	116 474	≤5	0.75 (0.31-1.80)	.52
Other	18 032	195 487	14	1.22 (0.72-2.06)	.46

Abbreviation: NA, not available.

^a^ All models were adjusted for maternal age, educational level, parity, cohabitation during pregnancy, any diabetes during pregnancy, birth year, and birth season.

^b^ Effect sizes cannot be estimated or were not shown due to the small number of cases.

^c^ Not including leukemia.

### Validation Using Swedish Registry Data

The patterns of association of maternal infections with any leukemia in Sweden were similar to those in Denmark, although the CIs of the effect estimates were wide in the Swedish data (eTable 11 in Supplement 1). Interaction analysis showed no evidence that the associations differed between the 2 countries.

### Updated Meta-analysis

To summarize all available evidence, we performed an updated meta-analysis of the association between maternal infection and childhood leukemia (eTable 12 in Supplement 1). When only cohort data (ie, the present study and the I4C analysis) were included in the meta-analysis, maternal urinary tract infection, but not respiratory tract infection, was associated with a higher risk of childhood leukemia. Adding previous case-control studies decreased risk associated with urinary tract infection but increased risk associated with respiratory tract infection. The meta-analysis of the current study and a previous case-control study suggested that genital tract infection was associated with a higher risk of childhood leukemia. Overall, this evidence suggests that several maternal infections are associated with a higher risk of childhood leukemia.

## Discussion

In this population-based cohort study, maternal infections during pregnancy were associated with a higher risk of childhood leukemia but not other types of childhood cancer. This association was mainly attributed to urinary and genital tract infections. The results of the sibling analysis suggest that this association was not likely to have been confounded by familial factors.

Both the present analysis of approximately 2.2 million Danish children and the I4C analysis^12^ of 310 000 children showed an association between maternal urinary tract infection and childhood leukemia. This consistency enhances confidence in this finding. However, it differs from the findings in 3 previous case-control studies, in which no association was observed.^11^ Of note, 2 studies used self-reported infection data and reported odds ratios (ORs) of 0.68 (95% CI, 0.42-1.09)^16^ and 0.56 (95% CI, 0.15-2.16),^23^ respectively. The third study used medical record data and reported an OR of 1.27 (95% CI, 0.69-2.34).^24^

We observed that genital tract infection was positively associated with childhood leukemia. This finding corroborates the results from a population-based case-control study in Sweden, in which a positive association was also reported between hospital-diagnosed lower genital tract infection and childhood leukemia.^24^ Another study in the US also reported a positive association for self-reported sexually transmitted infections.^16^ By contrast, the recent I4C analysis showed no association for self-reported vaginal thrush (nonsexually transmitted infection).^12^ We speculate that commensal organisms, such as vaginal thrush, are less likely to affect the fetus and less likely to contribute to childhood leukemia compared with sexually transmitted infections.

Respiratory tract infection was associated with a higher risk of childhood leukemia in the I4C analysis,^12^ but no association was observed in the present analysis. One reason for this discrepancy could be the differences in the infection assessment between the 2 analyses. Maternal infection was based on hospital diagnoses in the present study, whereas infections were primarily self-reported in the I4C cohorts; this yielded a substantial difference in the prevalence of respiratory tract infection. For example, the prevalence of hospital-diagnosed respiratory tract infection was 0.3% in the present study, whereas the prevalence of self-reported respiratory tract infection was greater than 30% in the I4C analysis. The 2 exposure definitions may represent infections with different severity; therefore, the results of the 2 studies are not directly comparable.

Genitourinary tract infection is common during pregnancy and has been considered a risk factor for adverse perinatal outcomes such as preterm birth. Pregnant women in many countries are tested for urinary tract infection and bacterial vaginosis (if symptomatic) and treated with antibiotics in antenatal care practice. We found that urinary and genital tract infection during pregnancy was associated with a higher risk of childhood leukemia, but the associated absolute risk remained small given the rarity of childhood leukemia. Future epidemiologic studies in different regions and mechanistic research are needed to confirm our findings and investigate the underlying mechanisms.

The lymphoblastic (ALL) and myeloid (AML) forms of childhood leukemia are considered to have distinct etiologies. Our results suggest similarities in their association with maternal infection, indicating common mechanisms with regard to these exposures. Both ALL and AML are regarded to have a prenatal origin, given evidence from the identification of leukemia-initiating translocations in neonatal blood spots from children who later develop ALL or AML.^25^ The structure of these prenatal translocations both for ALL and AML suggest nonspecific chromosomal breakage, followed by nonhomologous end-joining DNA repair^26^; such breakage and repair would be compatible with causation from inflammatory mechanisms surrounding infection and infection clearance. The commonality of mechanisms in ALL and AML is not unprecedented, as previous studies have shown that postnatal infections^27^ and breastfeeding^28^ were associated with both childhood ALL and AML. Considering the range of infectious vectors and the proximity of the infection to the developing fetus, the mechanisms for maternal infection are likely to be nonspecific and related to enhancement of localized inflammation, inflammatory mediators, and damage from reactive oxygen species.^29^

### Limitations

Although our cohort study has a number of strengths—such as the large sample size, high-quality medical registry data with prospective collection and national coverage, complete follow-up for cancer outcomes, and use of sibling analysis—several limitations should be acknowledged. Data on maternal infection were extracted from hospital diagnoses; thus, milder infections and those not diagnosed or treated in specialized health care facilities were not captured. Also, some infections could be captured because the mother sought care for other, more serious conditions, which might bias the association of maternal infections and childhood leukemia. However, controlling for maternal comorbidities in our analysis did not substantially change the results. In addition, outpatient data were available only from 1995 onward in Denmark. This might have led to inconsistency in the assessment of exposures in different periods. However, our stratified analysis revealed no significant differences in the results between the periods with and without outpatient data. Data on the treatments administered in the hospital for maternal infection were not available in our databases; thus, we were unable to investigate whether treatments modify the risk of childhood leukemia in offspring. Finally, although sibling analysis controlled for shared familial factors between siblings, residual confounding due to factors that differed between pregnancies is still possible.

## Conclusions

The findings of this large population-based cohort study suggest that maternal urinary and genital tract infections during pregnancy are associated with a higher risk of childhood leukemia in offspring. Given that little is known about the etiology of childhood leukemia, these findings suggest an important direction for research on the etiology of childhood leukemia as well as development of potential preventive measures.

## Notes

Supplement 1. **eMethods 1.** Descriptions of Danish National Registries
eReferences
**eMethods 2.** Description of Swedish Patient Registry Data Included in This Study **eTable 1.** Relevant Variables to This Study From National Registries in Denmark **eTable 2.** *ICD* Codes Used to Define Each Cancer Outcome **eTable 3.** *ICD* Codes for 386 Specific Types of Infections Used to Define Infection Exposures in Denmark **eTable 4.** *ICD* Codes Used to Define Comorbidities During Pregnancy **eTable 5.** *ICD* Codes Used to Identify Patients for the Subset of Swedish Patient Registry Data **eTable 6.** Associations of Maternal Infections at Each Trimester and Childhood Leukemia **eTable 7.** Associations of Maternal Infections and Childhood Leukemia Stratified by Periods With and Without Outpatient Infection Data or by Periods With Different Versions of *ICD* Codes **eTable 8.** Hazard Ratios for Each Infection From Models Without and With Additional Adjustment for Maternal Smoking During Early Pregnancy or Prepregnancy Body Mass Index **eTable 9.** Adjustment for Maternal Comorbidities During Pregnancy for the Association Between Maternal Infections and Any Childhood Leukemia **eTable 10.** Hazard Ratios for Each Infection After Excluding Children Born Preterm **eTable 11.** Associations of Maternal Infections and Childhood Leukemia Stratified by Country **eTable 12.** An Updated Meta-analysis for Maternal Infections During Pregnancy and Childhood Leukemia **eFigure 1.** Flowchart of Population Inclusion **eFigure 2.** Association of Any Maternal Infection and Specific Types of Infections During Pregnancy With Leukemia Subtypes (Acute Lymphoblastic Leukemia and Acute Myeloid Leukemia)

Supplement 2.
Data Sharing Statement
