# Supplementary material for: Evaluation of Maternal Infection During Pregnancy and Childhood Leukemia Among Offspring in Denmark
Source: JAMA Netw Open. 2023 Feb 20;6(2):e230133. doi: 10.1001/jamanetworkopen.2023.0133 (PMC9941888; doi:10.1001/jamanetworkopen.2023.0133)
Supplement: Supplement 1. — eMethods 1. Descriptions of Danish National Registries eReferences eMethods 2. Description of Swedish Patient Registry Data Included in This Study eTable 1. Relevant Variables to This Study From National Registries in Denmark eTable 2. ICD Codes Used to Define Each Cancer Outcome eTable 3. ICD Codes for 386 Specific Types of Infections Used to Define Infection Exposures in Denmark eTable 4. ICD Codes Used to Define Comorbidities During Pregnancy eTable 5. ICD Codes Used to Identify Patients for the Subset of Swedish Patient Registry Data eTable 6. Associations of Maternal Infections at Each Trimester and Childhood Leukemia eTable 7. Associations of Maternal Infections and Childhood Leukemia Stratified by Periods With and Without Outpatient Infection Data or by Periods With Different Versions of ICD Codes eTable 8. Hazard Ratios for Each Infection From Models Without and With Additional Adjustment for Maternal Smoking During Early Pregnancy or Prepregnancy Body Mass Index eTable 9. Adjustment for Maternal Comorbidities During Pregnancy for the Association Between Maternal Infections and Any Childhood Leukemia eTable 10. Hazard Ratios for Each Infection After Excluding Children Born Preterm eTable 11. Associations of Maternal Infections and Childhood Leukemia Stratified by Country eTable 12. An Updated Meta-analysis for Maternal Infections During Pregnancy and Childhood Leukemia eFigure 1. Flowchart of Population Inclusion eFigure 2. Association of Any Maternal Infection and Specific Types of Infections During Pregnancy With Leukemia Subtypes (Acute Lymphoblastic Leukemia and Acute Myeloid Leukemia) [file jamanetwopen-e230133-s001.pdf]

Supplementary Online Content

He JR, Yu Y, Fang F, et al. Evaluation of maternal infection during pregnancy and childhood leukemia among offspring in Denmark. *JAMA Netw Open*. 2023;6(2):e230133. doi:10.1001/jamanetworkopen.2023.0133

eMethods 1. Descriptions of Danish National Registries
eReferences
eMethods 2. Description of Swedish Patient Registry Data Included in This Study
eTable 1. Relevant Variables to This Study From National Registries in Denmark
eTable 2. *ICD* Codes Used to Define Each Cancer Outcome
eTable 3. *ICD* Codes for 386 Specific Types of Infections Used to Define Infection Exposures in Denmark
eTable 4. *ICD* Codes Used to Define Comorbidities During Pregnancy
eTable 5. *ICD* Codes Used to Identify Patients for the Subset of Swedish Patient Registry Data
eTable 6. Associations of Maternal Infections at Each Trimester and Childhood Leukemia
eTable 7. Associations of Maternal Infections and Childhood Leukemia Stratified by Periods With and Without Outpatient Infection Data or by Periods With Different Versions of *ICD* Codes
eTable 8. Hazard Ratios for Each Infection From Models Without and With Additional Adjustment for Maternal Smoking During Early Pregnancy or Prepregnancy Body Mass Index
eTable 9. Adjustment for Maternal Comorbidities During Pregnancy for the Association Between Maternal Infections and Any Childhood Leukemia
eTable 10. Hazard Ratios for Each Infection After Excluding Children Born Preterm
eTable 11. Associations of Maternal Infections and Childhood Leukemia Stratified by Country
eTable 12. An Updated Meta-analysis for Maternal Infections During Pregnancy and Childhood Leukemia
eFigure 1. Flowchart of Population Inclusion
eFigure 2. Association of Any Maternal Infection and Specific Types of Infections During Pregnancy With Leukemia Subtypes (Acute Lymphoblastic Leukemia and Acute Myeloid Leukemia)

This supplementary material has been provided by the authors to give readers additional information about their work.

© 2023 He JR et al. *JAMA Network Open*.

eMethods 1. Descriptions of Danish National Registries

Danish Medical Birth Registry

The Danish Medical Birth Registry was originally founded in 1968 and computerised in 1973.[1, 2] It covers all births in Denmark. Data were collected from the National Patient Registry, Danish Civil Registration System and separate forms on home births and stillbirths. Data include pregnancy-related background characteristics (e.g. parity, BMI, smoking, pregnancy complications) and delivery (e.g. delivery mode, date) and birth characteristics (e.g. sex, gestational age, Apgar score, birth weight).

Danish National Patient Registry

The Danish National Patient Registry was founded in 1977 and reached complete national coverage since 1978.[3] Data were collected from all hospitals in Denmark, including administrative data, diagnoses, treatments, and examinations. Diagnosis data are registered when the patient is discharged from the hospital or when an outpatient contact ends. Inpatient data have been collected since 1977, while outpatient data are available from 1995. For each hospital contact, a primary diagnosis is recorded along with optional secondary diagnoses. Danish International Classification of Diseases, 8th version (ICD-8) was used to code the diagnosis data during 1977-1993, and ICD-10 was adopted from 1994.

Danish National Cancer Registry

The Danish Cancer Registry was founded in 1942 and began systematic data collection on all incident malignant neoplasms (and certain precancerous and benign lesions) from 1943.[4] Available data include personal characteristics (e.g. sex, age at diagnosis) and tumour characteristics (e.g. diagnosis, date, topography, morphology, behaviour). The diagnosis is classified according to ICD-7 during 1943-1978 and ICD-10 after 1978 (converted from ICD-7 during 1978-2003).

Other nationwide registries

Other relevant Danish registries include: (1) Danish Civil Registration System, which collects information on all alive residents in Denmark since 1986.[5] Data include the unique personal identification number, name, sex, birth date, birthplace, marital status, identity of kinship (parents, spouse, children, siblings), vital status (e.g. migration, death), etc. (2) Danish Registry of Causes of Death, which registers all deaths (date and cause of death) among citizens in Denmark since 1875 and has been computerised since 1970.[6] (3) Integrated Database for Labour Market Research, which covers information on education, employment and Income since 1980.[7] (4) The Danish National Diabetes Registry,[8] which was established in 2006 and identifies diabetes patients using data on prescriptions with antidiabetic drugs in the National Prescription Registry and diagnoses of diabetes in the National Patient Registry and receipt of chiropody for diabetic patients in National Health Service Registry.

eReferences

1. Knudsen LB, Olsen J. The Danish Medical Birth Registry. Dan Med Bull. 1998; **45**(3): 320-3.
2. Bliddal M, Broe A, Pottegard A, Olsen J, Langhoff-Roos J. The Danish Medical Birth Register. European journal of epidemiology. 2018; **33**(1): 27-36.
3. Schmidt M, Schmidt SA, Sandegaard JL, Ehrenstein V, Pedersen L, Sorensen HT. The Danish National Patient Registry: a review of content, data quality, and research potential. Clinical epidemiology. 2015; **7**: 449-90.
4. Gjerstorff ML. The Danish Cancer Registry. Scand J Public Health. 2011; **39**(7 Suppl): 42-5.
5. Schmidt M, Pedersen L, Sorensen HT. The Danish Civil Registration System as a tool in epidemiology. European journal of epidemiology. 2014; **29**(8): 541-9.
6. Helweg-Larsen K. The Danish Register of Causes of Death. Scand J Public Health. 2011; **39**(7 Suppl): 26-9.
7. Timmermans B. The Danish integrated database for labor market research: towards demystification for the English speaking audience: Danish Research Unit for Industrial Dynamics; 2010.
8. Green A, Sortso C, Jensen PB, Emneus M. Validation of the danish national diabetes register. Clinical epidemiology. 2015; **7**: 5-15.

© 2023 He JR et al. *JAMA Network Open*.

eMethods 2. Description of Swedish Patient Registry Data Included in This Study

The Figure below shows the flowchart for Swedish data inclusion in the present study. For the Swedish patient registry data, only a subset was obtained for patients with specific diagnostic codes. These codes cover both infectious conditions (i.e. respiratory, neurological, cardiovascular infections, gastroenteritis and colitis, sepsis) or some non-communicable diseases (such as hypertension, diabetes, obesity, asthma, mental disorders) (Tables S5). A patient with any of these codes was eligible for inclusion in the subset. For the eligible patients, complete diagnostic codes during at same hospital visit were also available.

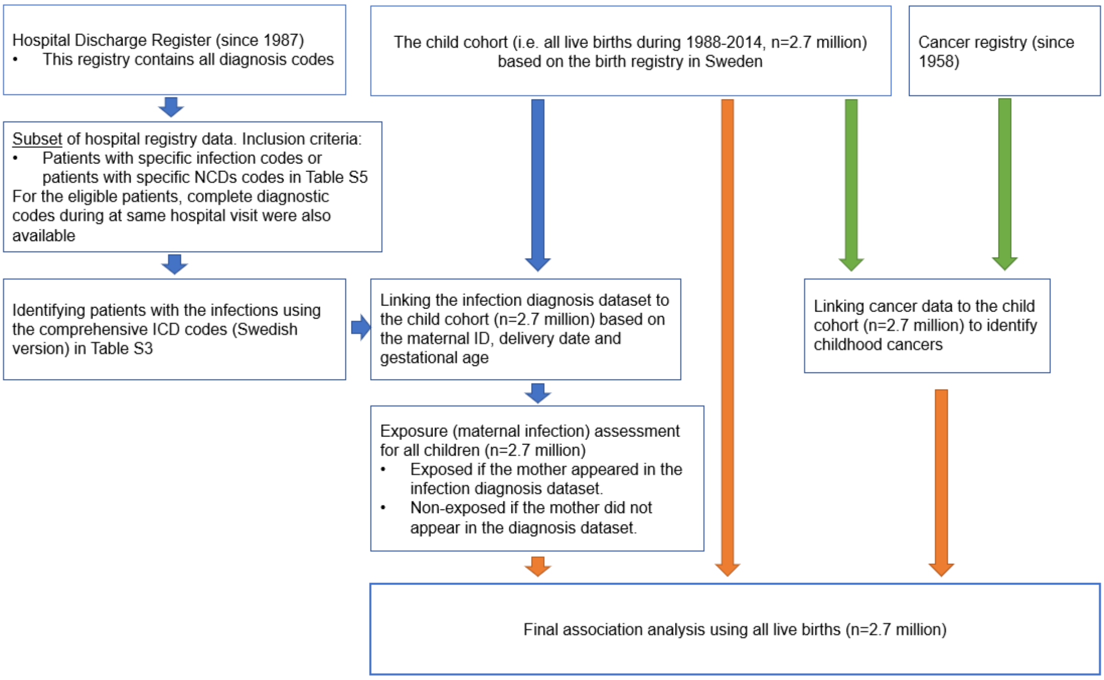

Flowchart of Swedish data inclusion.
NCD, non-communicable diseases. Blue arrows present the infection data inclusion; green ones represent cancer outcome ascertainment; orange ones represent data inclusion for the final association analysis. * Infections include respiratory infections (ICD-10: J00–J06, J12-J16), sepsis (A40-A41, O85), gastroenteritis and colitis (A09), neurological infections (A39.0, A39.2, A80-A89, G00- G02), any infection of the circulatory system (all related among I00-I99). Corresponding ICD-9 codes were also included. # NCDs include diabetes (ICD-10: E10-E14), hypertensive disorders in pregnancy (O10-O16), obesity (E66), Asthma (J45), rheumatoid arthritis (M05-M09), diseases of the circulatory system (I00-I99), mental disorders (F00-F99), congenital malformations (Q00-Q99) etc. Corresponding ICD-9 codes were also included.

Using this subset of Swedish patient registry data, we assessed maternal infection exposure for Sweden based on the comprehensive list of ICD codes in Table S3. Potential biases could result from this step. Some infections, such as genitourinary infections, were underdiagnosed because they were not listed in the inclusion criteria when the subset data were drawn. These infections could only be captured if the same patient had comorbidities listed in the inclusion criteria. We drew a directed acyclic graph (Figure below) to explore the bias caused by this, taking genitourinary infections as an example. Due to the inclusion criteria, mothers with morbidities were included in the subset, and thus they had a higher chance of being found to have genitourinary infections. By contrast, mothers without the morbidities were not included in the subset and were automatically categorised as having no genitourinary infections. Therefore, a mother with recorded morbidities would be more likely to be categorised into the infection exposure group than one without those morbidities. If these morbidities are risk factors for childhood leukaemia, a spurious association would be created between

© 2023 He JR et al. *JAMA Network Open*.

genitourinary infections and childhood leukaemia. Due to this potential limitation, we only used the Swedish dataset as a validation of the main findings in the Danish dataset.

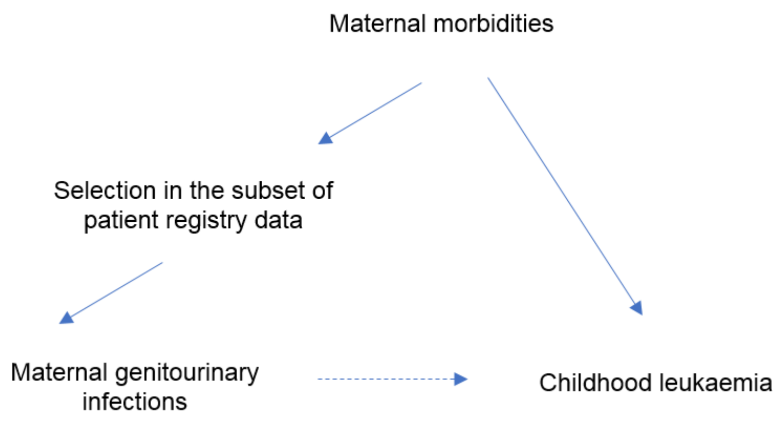

A directed acyclic graph showing the relation between maternal morbidities, selection for the subset data, genitourinary infections and childhood leukaemia

© 2023 He JR et al. *JAMA Network Open*.

eTable 1. Relevant Variables to This Study From National Registries in Denmark

National registry	Year covered	Variables relevant to the current study
Medical Birth Registry	Since 1968	Livebirth, singleton, sex, birth date, gestational age, birth weight, delivery mode, smoking during pregnancy, pre-pregnancy BMI, parity
National Hospitalization Registry	Inpatients, since 1977<br>Outpatients, since 1995	Admission date, discharge diagnosis used to extract infection variables and Down Syndrome (ICD-10 code, Q90; ICD-8 codes, 31050-31051, 31150-31151, 31250-31251, 31350-31351, 31450-31451, 31550-31551, and 75930-75939)
Cancer Registry	Since 1943	Date and diagnosis (used to extract outcome variables)
National Diabetes Registry	Since 1977*	Maternal diabetes before delivery
Civil Registration System	Since 1968	Birthdate, emigration, marital status, identity of kinship
Registry of Causes of Death	Since 1970	Date of death
Integrated Database for Labour Market Research	Since 1980	Education level

* Danish National Diabetes Registry was established in 2006 and source data were from several health registries which collected data from 1977 at the earliest.

© 2023 He JR et al. *JAMA Network Open*.

eTable 2. *ICD* Codes Used to Define Each Cancer Outcome

Cancer type	ICD-10
Leukaemia	C91-95
ALL	C91.0
AML	C92.0
Brain and central nervous system tumours	C70-72, D32, D33, D35.2, D35.3, D35.4 D42, D43, D44.3-D44.5
Lymphomas	C81-C86, C96
Other types	C00-97 except above
Any cancer	C00-97

© 2023 He JR et al. *JAMA Network Open*.

eTable 3. *ICD* Codes for 386 Specific Types of Infections Used to Define Infection Exposures in Denmark

Infection variables	ICD-10	ICD-8
**Any infection**	Any below	Any below
**Infections by site**		
Respiratory tract infections	J00-J06, J09-J18, J20-J22, A15-16, A20.2, A21.2, A22.1, A31.0, A37, A38, A42.0, A48.1, B00.2, B01.2, B05.2, B25.0, B27, B37.1, B38.0, B38.1, B38.2, B39.0-B39.2, B40.0-B40.2, B41.0, B42.0, B44.0-B44.2, B45.0, B46.0, B58.3, B59, B67.1, J32, J34.0, J35.0, J36, J37, J383C, J383D, J387F, J387G, J39.0-39.1, J398A, J85-J86.	460-466, 470-474, 480-486, 01099, 011, 012,02019, 033, 034, 075, 12219, 503, 50002, 501, 50699, 50800-50803, 513, 510, 511.
Urinary tract infections	N30 (except N30.4), N39.0, O23.0-O23.4, N33.0, N29.1, N370A, N10–N12, N13.6, N15.1, N15.9, N16.0, N34.0-N34.1	590, 595 (except 59504), 59906, 635, 01600, 01601, 590, 597
Genital tract infections	A50-A64, N70-76, N77.0, N77.1, O23.5, O23.9, B37.3, O07.0, O07.5<br>Sexually transmitted infections: A50-A64	05402, 090-099, 131, 612–614, 616.0, 620, 622, 62949, 630, 01606, 01608, 01609<br>Sexually transmitted infections: 05402, 090-099, 131
Digestive system infections	A00-A09, B15-B19, A18.3, A22.2, B26.3, B67.0, B67.5, B67.8, B71, B81, B82, B58.1, K04.7, K04.6, K04.0, K05.2, K10.2, K11.3, K112A, K112C, K12.0, K12.1, K12.2, K130A, K140A, K14.0, K209A, K23.0, K35, K57.0, K57.2, K57.4, K57.8, K61, K628N, K65.0, K67, K63.0, K75.0, K77.0, K81.0, K85, K871A, K871B, K93.0	000-009, 070, 014, 07201, 123, 127, 129, 52259, 52209, 52339, 52649, 52739, 52722, 52720, 5280-5282, 52838-52839, 5290, 540, 566, 56700-56702, 56708, 57201, 57209, 57500, 57703, 57708, 57709
Others	Sepsis: A40, A41, A02.1, A22.7, A26.7, A32.7, A39.2, A39.3, A39.4, A42.7, B37.7, A20.7, A48.3, R57.2, O75.3, J950A	Sepsis: 038, 03610, 03611, 67002, 67003, 67008, 67009

© 2023 He JR et al. *JAMA Network Open*.

	Central nervous system: A80-A89, G00-G02, G04.2, G05.0-G05.2, G06, G07, A06.6, A17, A20.3,A22.8, A32.1, A39.0, A50.4, A52.1, A52.2, A52.3, B00.3, B00.4, B01.0, B01.1, B02.0, B02.1, B05.0, , B05.1, B06.0, B26.1, B26.2, B58.2, B37.5, B38.4, B43.1, B45.1, B46.1, B50.0, B57.4, B60.2, B69.0, B83.2, I02	Central nervous system: 040-046, 320, 322, 32300, 013, 02701, 03609, 03610, 09049, 094, 062-065, 07199, 05403, 05201, 05302, 05501, 05601, 07202, 07501, 07929, 392
**eTable 3. continued.**		
**Infection variables**	**ICD-10**	**ICD-8**
Others (continued)	Skin: B00-B09, L00-L08, A18.4, A22.0, A26.0, A31.1, A32.0, A36.3, B35, B36, B37.2, B43.0, B43.2, B45.2, B46.3, B55.1, B65.3, L30.3, L70.0	Skin: 050-057, 680-686, 0170, 110-111, 08519, 08529, 12039, 70609, 70610
	Eye: H00.0, H01.0, H03.0, H03.1, H04.3, H05.0, H06.1, H10.0, H10.2-H10.5, H10.8, H10.9, H13.0, H13.1, H16.2, H16.3, H16.9, H19.0, H19.1, H19.2, H22.0, H32.0, H44.0, H44.1	Eye: 362, 36100-36101, 36108-36109, 36800, 36805, 36900, 36901, 360 (except 36003), 36391, 36399, 05404, 05300, 09039, 09821, 09828, 09829, 36600
	Ear: H60.0, H60.1, H60.3, H62.0, H62.1, H62.2, H62.3, H65.0, H65.1, H66, H67.0, H67.1, H70.0, H70.1, H75.0, H94.0	Ear: 380, 381 (except 38102), 382, 38309, 38319
	Musculoskeletal system: M00, M01, M46.3, M46.5, M49.0, M49.1, M49.2, M49.3, M60.0, M63.0, M63.1, M63.2, M65.0, M65.1, M68.0, M71.0, M71.1, M73.0, M73.1, M86, M90.0, M90.1, M90.2	Musculoskeletal system:710, 73299, 72000-72029, 72031, 015
	Circulatory system: I00, I01, I30, I32.0, I32.1, I33, I398A, I398B, I398D, I398F, I398G, I398H, I40.0, I41.0, I41.1, I43.0, I52.0, I52.1	Circulatory system: 390, 391, 420, 07429, 093, 421, 422

© 2023 He JR et al. *JAMA Network Open*.

	Other sites: D73.3, N61, O98, O35.3, O358B, O41.1, T79.3, T80.2, T81.4, T82.6, T82.7, T83.5, T83.6, T84.5, T84.6, T84.7, T88.0, E06.0, E32.1; and others in A00–B99	Other sites: 28940, 61100, 61101, 763, 76109, 76129, 76139, 76149, 99939, 99859, 24500, 24501; and others in 000–136

© 2023 He JR et al. *JAMA Network Open*.

eTable 4. *ICD* Codes Used to Define Comorbidities During Pregnancy

Comorbidities	ICD-10	ICD-8
Hypertensive disorders	I10-I13, I15, O10- O16	400, 40199, 63700, 63703, 63704, 63709, 63719, 76029
Anaemia	D50-D53, D55-D59, D60-D64, O990	280–285, 633
Obstetric haemorrhage	O20, O44.1, O45, O46	632, 7700, 7701
Hyperemesis	O21	63809, 63899, 76249
Asthma	J45	493

© 2023 He JR et al. *JAMA Network Open*.

eTable 5. *ICD* Codes Used to Identify Patients for the Subset of Swedish Patient Registry Data[#]

Categories of diagnosis	ICD-10	ICD-9
**Infection-related diagnosis**		
Respiratory tract infections	J00–J06, J12-J16	460–466, 480, 481, 482
Urinary tract infections	None	None
Genital tract infections	None	None
Digestive system infections	A09	009
Other infections	Sepsis: A40, A41, A39.2, O75.3<br>Central nervous system: A39.0, A80-A89, G00, G01, G02<br>Musculoskeletal system: M46.3, M46.5<br>Circulatory system: I00, I01, I30, I32.0, I32.1, I33, I40.0, I41.0, I41.1, I43.0, I52.0, I52.1	Sepsis: 038<br>Central nervous system: 045, 046<br>Musculoskeletal system: 711A, 711E, 711F, 711G, 711H, 711W<br>Circulatory system: 420, 421, 422<br>Other sites: 040, 041
**Non-infection diagnosis**		
Diabetes mellitus	E10-E14	250
Hypertension and circulatory system	O10-O16, I00-I99*****	642, 390-398, 401-405, 410-417, 423-438, 440-448, 451-459
Obesity	E66	278
Asthma	J45	493
Congenital anomalies	Q00-Q99	740-759
Arthropathies-related	M05-M13 (except M10), M25	710-719 (except 717-718)
Mental disorders	F00-F99	290-319
Various accidents or injuries	V01-Y98	E807-E999
Others	O22, O74, O75*****, O85-O90, O99, J46, L40, M32, M45, M46*****	648, 666, 668-674, 696, 711*****, 720, 726, 734

[#] A patient with any codes in the table was eligible for inclusion in the subset. For the eligible patients, complete diagnostic codes during at same hospital visit were also available.
* Excluding infection-related diagnosis

© 2023 He JR et al. *JAMA Network Open*.

eTable 6. Associations of Maternal Infections at Each Trimester and Childhood Leukemia

Infection during pregnancy	Exposure timing [a]	HR (95% CI)	P for heterogeneity
Any infection	1st trimester	1.36 (0.82, 2.27)	0.17
	2nd trimester	1.90 (1.30, 2.79)	
	3rd trimester	1.12 (0.74, 1.67)	
Genitourinary tract infection	1st trimester	1.97 (1.14, 3.42)	0.34
	2nd trimester	2.29 (1.47, 3.57)	
	3rd trimester	1.43 (0.91, 2.25)	
Urinary tract infection	1st trimester	1.74 (0.87, 3.50)	0.36
	2nd trimester	1.99 (1.12, 3.51)	
	3rd trimester	1.13 (0.64, 1.99)	
Genital tract infection	1st trimester	2.40 (1.00, 5.79)	0.76
	2nd trimester	3.54 (1.90, 6.60)	
	3rd trimester	2.80 (1.40, 5.62)	

[a] Women could be repeatedly infected during different trimesters.

HR, hazard ratio; CI, confidence interval. All models were adjusted for maternal age, educational level, parity, cohabitation during pregnancy, any diabetes during pregnancy, birth year and birth season.

© 2023 He JR et al. *JAMA Network Open*.

eTable 7. Associations of Maternal Infections and Childhood Leukemia Stratified by Periods With and Without Outpatient Infection Data or by Periods With Different Versions of *ICD* Codes

Infection during pregnancy	Period	HR (95% CI)	$P_{interaction}$
Stratified by periods with and without outpatient infection data			
Any infection	Only inpatient data	1.36 (0.90, 2.07)	1.00
	Plus outpatient data	1.33 (0.94, 1.89)	
Genito-urinary tract infection	Only inpatient data	1.73 (1.05, 2.85)	0.92
	Plus outpatient data	1.73 (1.18, 2.53)	
Urinary tract infection	Only inpatient data	1.48 (0.76, 2.85)	0.65
	Plus outpatient data	1.71 (1.12, 2.63)	
Genital tract infection	Only inpatient data	3.03 (1.62, 5.66)	0.37
	Plus outpatient data	1.88 (0.89, 3.96)	
Respiratory tract infection	Only inpatient data	0.60 (0.08, 4.27)	0.69
	Plus outpatient data	0.96 (0.24, 3.85)	
Digestive system infection	Only inpatient data	1.16 (0.37, 3.60)	0.56
	Plus outpatient data	0.66 (0.17, 2.65)	
Other infections	Only inpatient data	1.38 (0.62, 3.08)	0.22
	Plus outpatient data	0.57 (0.18, 1.76)	
Stratified by periods with different versions of ICD codes			
Any infection	ICD-8	1.33 (0.86, 2.05)	0.89
	ICD-10	1.36 (0.97, 1.90)	
Genito-urinary tract infection	ICD-8	1.84 (1.12, 3.03)	0.82
	ICD-10	1.67 (1.14, 2.45)	
Urinary tract infection	ICD-8	1.58 (0.82, 3.06)	0.85
	ICD-10	1.67 (1.09, 2.56)	
Genital tract infection	ICD-8	3.17 (1.69, 5.93)	0.28
	ICD-10	1.81 (0.86, 3.82)	
Respiratory tract infection	ICD-8	0.64 (0.09, 4.53)	0.76
	ICD-10	0.92 (0.23, 3.68)	
Digestive system infection	ICD-8	0.82 (0.21, 3.30)	0.87
	ICD-10	0.94 (0.30, 2.93)	
Other infections	ICD-8	1.22 (0.51, 2.95)	0.44
	ICD-10	0.72 (0.27, 1.92)	

HR, hazard ratio; CI, confidence interval. All models were adjusted for maternal age, educational level, parity, cohabitation during pregnancy, any diabetes during pregnancy, birth year and birth season.

© 2023 He JR et al. *JAMA Network Open*.

eTable 8. Hazard Ratios for Each Infection From Models Without and With Additional Adjustment for Maternal Smoking During Early Pregnancy or Prepregnancy Body Mass Index

Adjustment factors	HR (95% CI)	
	Non-adjusted	Adjusted
Smoking during early pregnancy [a]		
Any infection	1.41 (1.03, 1.93)	1.41 (1.03, 1.94)
Genitourinary tract infection	1.77 (1.24, 2.53)	1.77 (1.24, 2.53)
Urinary tract infection	1.71 (1.14, 2.57)	1.71 (1.14, 2.57)
Genital tract infection	2.04 (1.05, 3.94)	2.05 (1.06, 3.97)
Respiratory tract infection	0.82 (0.20, 3.29)	0.82 (0.21, 3.30)
Digestive system infection	0.81 (0.26, 2.52)	0.81 (0.26, 2.53)
Other infections	0.97 (0.44, 2.17)	0.98 (0.44, 2.18)
Pre-pregnancy BMI [b]		
Any infection	1.27 (0.78, 2.08)	1.27 (0.78, 2.08)
Genitourinary tract infection	1.74 (1.05, 2.90)	1.74 (1.05, 2.90)
Urinary tract infection	2.09 (1.26, 3.48)	2.09 (1.26, 3.48)
Genital tract infection	0.57 (0.08, 4.10)	0.57 (0.08, 4.10)
Respiratory tract infection	1.02 (0.14, 7.31)	1.03 (0.14, 7.31)
Digestive system infection	–	–
Other infections	–	–

[a] Data on maternal smoking were available from 1991; [b] Data on pre-pregnancy BMI were available from 2004.

HR, hazard ratio; CI, confidence interval. All models were adjusted for maternal age, educational level, parity, cohabitation during pregnancy, any diabetes during pregnancy, birth year and birth season. –, effect sizes cannot be estimated or were not shown due to the small number of cases

© 2023 He JR et al. *JAMA Network Open*.

eTable 9. Adjustment for Maternal Comorbidities During Pregnancy for the Association Between Maternal Infections and Any Childhood Leukemia

Infection during pregnancy	HR (95% CI) of adjustment for each comorbidity					
	Hypertensive disorders	Anaemia	Haemorrhage	Hyperemesis	Asthma	Any comorbidities
Any infection	1.35 (1.03, 1.76)	1.34 (1.03, 1.75)	1.35 (1.04, 1.77)	1.35 (1.03, 1.76)	1.36 (1.04, 1.77)	1.34 (1.03, 1.75)
Genitourinary tract infection	1.73 (1.28, 2.34)	1.73 (1.28, 2.34)	1.74 (1.29, 2.35)	1.74 (1.29, 2.35)	1.74 (1.29, 2.36)	1.72 (1.27, 2.33)
Urinary tract infection	1.64 (1.15, 2.35)	1.64 (1.15, 2.35)	1.65 (1.15, 2.36)	1.65 (1.15, 2.36)	1.65 (1.15, 2.36)	1.64 (1.14, 2.34)
Genital tract infection	2.42 (1.50, 3.91)	2.41 (1.49, 3.90)	2.43 (1.50, 3.92)	2.43 (1.50, 3.92)	2.43 (1.50, 3.92)	2.41 (1.49, 3.90)
Respiratory tract infection	0.80 (0.26, 2.49)	0.80 (0.26, 2.48)	0.80 (0.26, 2.50)	0.80 (0.26, 2.50)	0.82 (0.26, 2.56)	0.80 (0.26, 2.48)
Digestive system infection	0.89 (0.37, 2.15)	0.89 (0.37, 2.14)	0.89 (0.37, 2.15)	0.90 (0.37, 2.16)	0.90 (0.37, 2.16)	0.89 (0.37, 2.15)
Other infections	0.93 (0.48, 1.80)	0.92 (0.48, 1.78)	0.93 (0.48, 1.80)	0.93 (0.49, 1.80)	0.94 (0.49, 1.80)	0.93 (0.48, 1.79)

HR, hazard ratio; CI, confidence interval. All models were adjusted for maternal age, educational level, parity, cohabitation during pregnancy, any diabetes during pregnancy, birth year and birth season.

© 2023 He JR et al. *JAMA Network Open*.

eTable 10. Hazard Ratios for Each Infection After Excluding Children Born Preterm

Infection variables	HR (95% CI)	P value
Any infection	1.33 (1.01, 1.77)	0.05
Genito-urinary tract infection	1.69 (1.23, 2.33)	0.001
Urinary tract infection	1.59 (1.09, 2.34)	0.02
Genital tract infection	2.30 (1.38, 3.83)	0.001
Respiratory tract infection	0.87 (0.28, 2.69)	0.80
Digestive system infection	0.96 (0.40, 2.31)	0.93
Other infections	0.94 (0.47, 1.88)	0.86

HR, hazard ratio; CI, confidence interval. All models were adjusted for maternal age, educational level, parity, cohabitation during pregnancy, any diabetes during pregnancy, birth year and birth season.

© 2023 He JR et al. *JAMA Network Open*.

eTable 11. Associations of Maternal Infections and Childhood Leukemia Stratified by Country

Infection during pregnancy	Country	Prevalence (%)	HR (95% CI)	$P_{interaction}$
Any infection	Sweden	1.79	1.34 (0.93, 1.93)	0.98
	Denmark	3.68	1.34 (1.03, 1.75)	
Genitourinary tract infection	Sweden	0.33	1.47 (0.66, 3.29)	0.77
	Denmark	2.28	1.72 (1.27, 2.32)	
Urinary tract infection	Sweden	0.18	1.11 (0.36, 3.45)	0.55
	Denmark	1.69	1.64 (1.15, 2.34)	
Genital tract infection	Sweden	0.15	2.12 (0.68, 6.58)	0.88
	Denmark	0.65	2.39 (1.48, 3.86)	
Respiratory tract infection	Sweden	0.50	1.49 (0.77, 2.87)	0.35
	Denmark	0.33	0.80 (0.26, 2.47)	
Digestive system infection	Sweden	0.31	1.29 (0.54, 3.10)	0.54
	Denmark	0.45	0.89 (0.37, 2.13)	
Other infections	Sweden	0.78	1.20 (0.66, 2.18)	0.57
	Denmark	0.81	0.93 (0.48, 1.79)	

HR, hazard ratio; CI, confidence interval. All models were adjusted for maternal age, educational level, parity, cohabitation during pregnancy, any diabetes during pregnancy, birth year and birth season.

© 2023 He JR et al. *JAMA Network Open*.

eTable 12. An Updated Meta-analysis for Maternal Infections During Pregnancy and Childhood Leukemia[a]

Characteristics	Data sources				Updated meta-analysis [f]	
	Denmark (current study)	I4C cohorts	Previous systematic review		Only cohort studies [g]	All studies
Urinary tract infection	1.65 (1.15-2.36)	1.68 (1.10-2.58)	0.84 (0.53-1.35) [b]		1.66 (1.26-2.99) I^2=0% [f]	1.35 (0.89-2.05) I^2= 67%
Genital tract infection	2.42 (1.50-3.92)	–	1.78 (1.17-2.72) [b, c, d]		–	2.04 (1.48-2.79) I^2=0% [f]
Sexually transmitted infection	3.13 (1.73-5.67)	–	7.59 (1.58-36.56) [b, c]		–	3.57 (1.93-6.63) I^2=7% [f]
Respiratory tract infection	0.80 (0.26-2.50)	1.54 (1.05-2.26)	1.46 (0.58–3.67) [b, c]		1.39 (0.87-2.22) I^2=13% [f]	1.44 (1.03-2.02) I^2=0% [f]

[a] Hazard ratio was used for the analyses for the current study and the I4C; odds ratio was used for previous systematic review.
[b] Only case-control studies; [c] Only one study; [d] Low genital tract infection; [e] Pooled odds ratio was 1.43 (0.93-2.20) for case-control studies and 4.82 (0.69-33.85) for cohort studies in the previous systematic review. [f] Random-effect model (Dersimonian-Laird method) for the meta-analysis, and I^2 was used to measure heterogeneity. [g] Including results of the current study, I4C data and cohort studies in the previous systematic review. –, effect estimates were unavailable.

© 2023 He JR et al. *JAMA Network Open*.

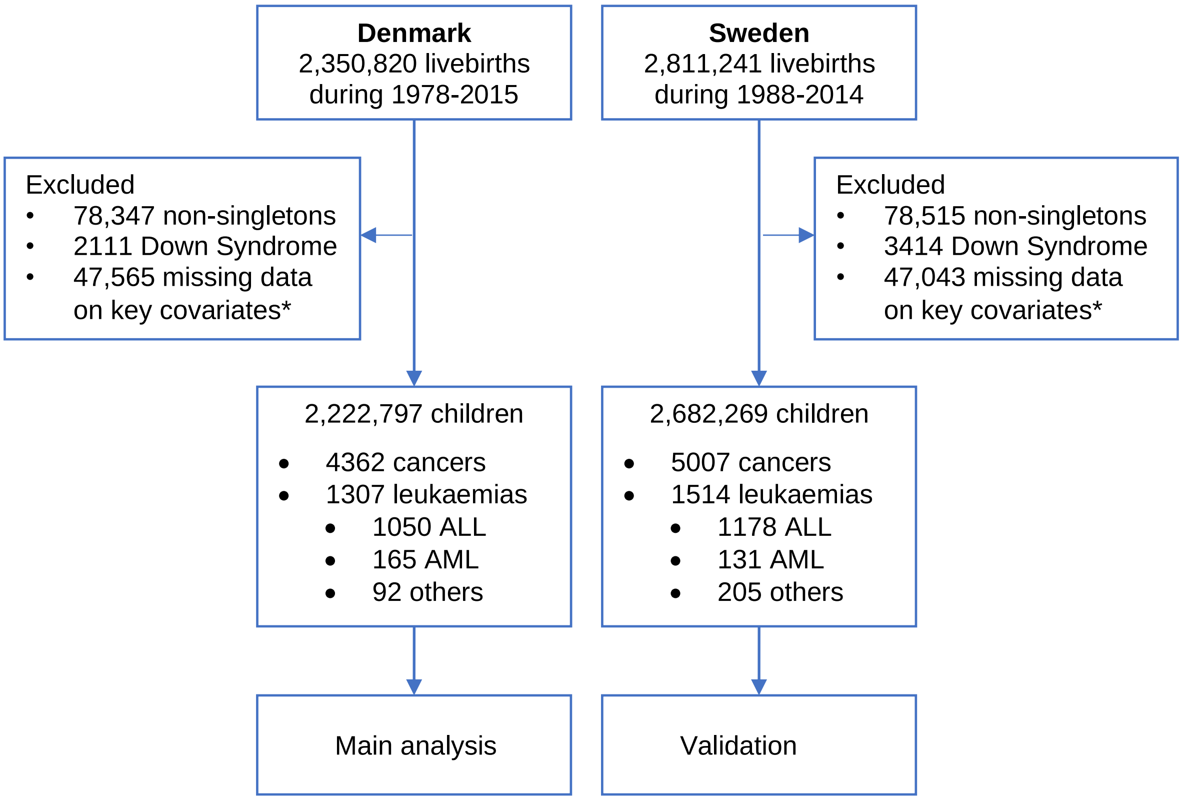

eFigure 1. Flowchart of Population Inclusion

*, key covariates refer to maternal age, education level, marital status, parity, diabetes.

© 2023 He JR et al. *JAMA Network Open*.

Infection variable	Acute lymphoblastic leukaemia n cases	Acute lymphoblastic leukaemia HR (95% CI)	Acute myeloid leukaemia n cases	Acute myeloid leukaemia HR (95% CI)
No infection	1008	1.00 (reference)	158	1.00 (reference)
Any infection	42	1.25 (0.92, 1.70)	7	1.35 (0.63, 2.88)
Infections by site				
Genitourinary tract infection	30	1.49 (1.04, 2.15)	7	2.24 (1.05, 4.81)
Urinary tract infection	20	1.34 (0.86, 2.09)	≤5	1.74 (0.64, 4.72)
Genital tract infection	12	2.15 (1.21, 3.80)	≤5	5.60 (2.29, 13.71)
Respiratory tract infection	≤5	0.67 (0.17, 2.67)	0	—
Digestive system infection	≤5	1.12 (0.46, 2.69)	0	—
Other infections	8	1.03 (0.52, 2.08)	0	—

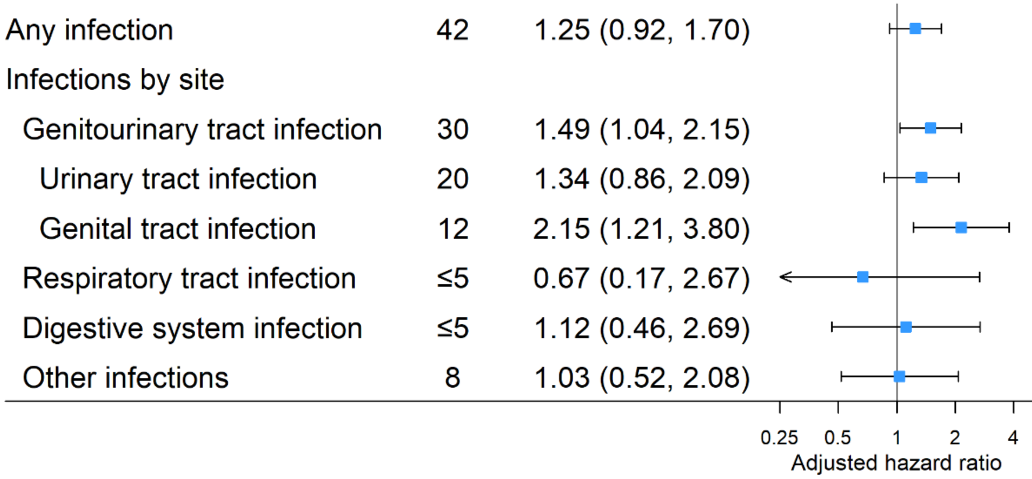

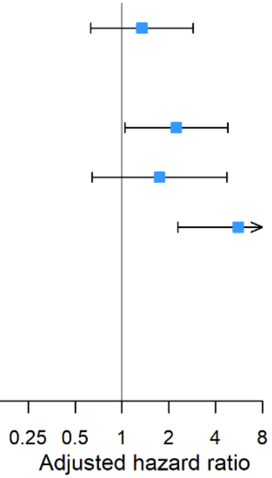

eFigure 2. Association of Any Maternal Infection and Specific Types of Infections During Pregnancy With Leukemia Subtypes (Acute Lymphoblastic Leukemia and Acute Myeloid Leukemia)

HR, hazard ratio; CI, confidence interval. All models were adjusted for maternal age, educational level, parity, cohabitation during pregnancy, any diabetes during pregnancy, birth year and birth season. –, effect sizes cannot be estimated or were not shown due to the small number of cases.

© 2023 He JR et al. *JAMA Network Open*.
