# Supplementary material for: Evaluation of Maternal Infection During Pregnancy and Childhood Leukemia Among Offspring in Denmark
Source: JAMA Netw Open. 2023 Feb 20;6(2):e230133. doi: 10.1001/jamanetworkopen.2023.0133 (PMC9941888; doi:10.1001/jamanetworkopen.2023.0133)
Supplement: Supplement 2. — Data Sharing Statement [file jamanetwopen-e230133-s002.pdf]

Data Sharing Statement

He. Evaluation of Maternal Infection During Pregnancy and Childhood Leukemia Among Offspring in Denmark. *JAMA Netw Open*. Published February 20, 2023. doi:10.1001/jamanetworkopen.2023.0133

Data
Data available: No

Additional Information
Explanation for why data not available: Due to the data regulation policy, data are not publicly available.
